# Was EU’s COVID-19 vaccine procurement strategy irrational? A re-analysis based on cost-effectiveness considerations

**DOI:** 10.1186/s12913-022-08726-4

**Published:** 2022-11-24

**Authors:** Afschin Gandjour

**Affiliations:** Frankfurt School of Finance & Management, Adickesallee 32-34, 60322 Frankfurt am Main, Germany

**Keywords:** European Union, COVID-19, Vaccine, Procurement, Cost-effectiveness

## Abstract

**Aim:**

The European Union (EU) has received criticism for being slow to secure coronavirus disease (COVID-19) vaccine contracts in 2020 before the approval of the first COVID-19 vaccine. This study aimed to retrospectively analyze the EU’s COVID-19 vaccine procurement strategy. To this end, the study retrospectively determined the minimum vaccine efficacy that made vaccination cost-effective from a societal perspective in Germany before clinical trial announcements in late 2020. The results were compared with the expected vaccine efficacy before the announcements.

**Methods:**

Two strategies were analyzed: vaccination followed by the complete lifting of mitigation measures and a long-term mitigation strategy. A decision model was constructed using, for example, information on age-specific fatality rates, intensive care unit costs and outcomes, and herd protection thresholds. The base-case time horizon was 5 years. Cost-effectiveness of vaccination was determined in terms of the costs per life-year gained. The value of an additional life-year was borrowed from new, innovative oncological drugs, as cancer is a condition with a perceived threat similar to that of COVID-19.

**Results:**

A vaccine with 50% efficacy against death due to COVID-19 was not clearly cost-effective compared with a long-term mitigation strategy if mitigation measures were planned to be lifted after vaccine rollout. The minimum vaccine efficacy required to achieve cost-effectiveness was 40% in the base case. The sensitivity analysis showed considerable variation around the minimum vaccine efficacy, extending above 50% for some of the input variables.

**Conclusions:**

This study showed that vaccine efficacy levels expected before clinical trial announcements did not clearly justify lifting mitigation measures from a cost-effectiveness standpoint. Hence, the EU’s sluggish procurement strategy still appeared to be rational at the time of decision making.

**Supplementary Information:**

The online version contains supplementary material available at 10.1186/s12913-022-08726-4.

## Introduction

In November 2020, the pharmaceutical companies Pfizer/BioNTech and Moderna independently announced that their vaccine candidates against SARS-CoV-2 demonstrated evidence of efficacy against coronavirus disease (COVID-19) in participants without prior evidence of SARS-CoV-2 infection. The case splits between vaccinated individuals and those who received the placebo indicated a vaccine efficacy rate above 90% [18, 35]. The European Commission approved the Pfizer/BioNTech and Moderna vaccines for use across 27 member states on December 21, 2020 and January 6, 2021, respectively. As of July 2022, the Commission has provided the conditional marketing authorization for six vaccines.

On June 17, 2020, the European Union (EU) proposed a strategy for the European Commission to centrally purchase COVID-19 vaccines on behalf of all EU countries [13]. Before the first approval, the EU Commission signed contracts with six vaccine manufacturers: Pfizer/BioNTech, Moderna, AstraZeneca, CureVac, Johnson & Johnson, and Sanofi. In total, almost 2 billion doses of vaccines were secured. This was basically enough for the 450 million inhabitants of the 27 EU member states, even if two doses per person had to be administered for almost all vaccines and not all vaccines would be approved. Nevertheless, the EU has received criticism for being slower than Israel, the United Kingdom, and the United States in securing vaccine contracts, thus slowing down vaccine rollout [5].

According to EU Commission spokesman Stefan De Keersmaecker, the EU wanted to position itself broadly. He argued that at that time there was no way of knowing which vaccine would be marketable first or at all [11]. The lower original order number of 200 million doses from Pfizer/BioNTech and 80 million doses from Moderna was partly due to their innovative technology and high prices. The Pfizer/BioNTech vaccine also has to be cooled to minus 70 °C and is therefore comparatively difficult to handle [11].

Although decisions on the EU’s vaccine procurement strategy were thus mainly based on criteria other than cost-effectiveness, cost-effectiveness should have been an important criterion given the scarcity of resources, particularly in times of gross domestic product (GDP) contraction. Therefore, the purpose of this study was to re-analyze the appropriateness of the EU’s vaccine procurement strategy based on the cost-effectiveness criterion. To this end, the present study retrospectively determined the minimum efficacy of a vaccine that was necessary to obtain an acceptable cost-effectiveness ratio in the general German population before the announcements of the first phase III trial results. The estimated minimum efficacy allows for a comparison with the anticipated efficacy levels before the announcement. For this purpose, the present study used the best available data from the second half of 2020, in accordance with the position to “evaluat[e] the currently available information in the best possible way” [23].

## Methods

### Conceptual approach

#### Cost-effectiveness

A new vaccine is considered to be cost-effective if its incremental cost-effectiveness ratio (ICER) versus a less effective treatment is smaller than or equal to the cost-effectiveness threshold λ:

1 $$\frac{c}{h}=\frac{v-s+b}{h}\le \uplambda,$$

where *c* is incremental costs; *h* denotes incremental net health benefits including harm from side effects; *v* is the cost of the new vaccine plus the costs of vaccine administration, government subsidies for vaccine research, scientific research failures, establishing vaccination centers, transportation, and managing side effects; *s* denotes savings from avoiding COVID-19-related morbidity; and *b* refers to the cost resulting from avoidance of COVID-19 death.

#### Minimum efficacy

As mentioned above, this study took the perspective before the approval of the first COVID-19 vaccine. The aim of this study was to determine the minimum vaccine efficacy against death due to COVID-19 that makes vaccination cost-effective. Replacing the unequal sign in Eq. (1) by an equal sign and rearranging Eq. (1) yields the minimum health benefit *h*_min_:

2 $$\frac{v-s+b}{\uplambda}={h}_{\textrm{min}}.$$

Next, *h*_min_ is converted into a minimum relative efficacy *e*_min_ compared with the maximum health benefit *h*_max_:

3 $${e}_{\textrm{min}}=\frac{h_{\textrm{min}}}{h_{\textrm{max}}},$$

where *h*_max_ equals mortality in the absence of vaccination.

#### Comparators

As a comparator of a COVID-19 vaccine, this study used a long-term mitigation strategy including partial lockdowns/shutdowns. This was the COVID-19 response strategy in Germany during the first pandemic wave. This study did not assume a suppression of the pandemic, however, because the strategy chosen by the German government leaned more toward mitigation than suppression. This mitigation strategy included compulsory face masks, physical distancing, and quarantine directives but also a shutdown of businesses such as nightclubs (in sum, a partial lockdown/shutdown).

#### Decision model

A decision model was constructed based on a previously developed and validated model [21]. The latter model determines the gain in life-years of a strategy that is successful in ‘squashing the curve’ compared with the situation before the pandemic. It is based a life-table model that summarizes the age-specific mortality impact of the COVID-19 pandemic. The base-case calculation relies on an independence assumption, implying that individuals not dying from COVID 19 have the same probability of death as all individuals before the rise of the pandemic. Given that patients who die from COVID 19 tend to have more comorbidities [49], a harvesting effect was assumed in a sensitivity analysis. This approach presumes that those who died from COVID-19 were sicker and would have died any-way. In this scenario, it was assumed that for age groups with excess mortality associated with COVID-19 (the difference between observed and pre-pandemic mortality rates) that except for COVID-19, there were no other causes of death in the forthcoming 12 months.

Given the absence of suppression, it was not assumed that further waves of the pandemic were prevented. Hence, the number of life-years gained from ‘squashing the curve’ were adjusted for the expected number of pandemic waves and the resulting death toll under mitigation. To this end, the death toll of the first pandemic wave in Germany (the termination was set to July 31, 2020) was multiplied by the expected number of pandemic waves and the resulting figure was subtracted from the gain in life-years by ‘squashing the curve’. Given that some commentators predicted the second wave to be substantially worse than the first, a doubling of the death toll was assumed in a sensitivity analysis.

There was no further adjustment of the number of life-years gained for a possible deferral of elective procedures, assuming that intensive care unit (ICU) capacity would be sufficient in future pandemic waves.

The time horizon (5 years in the base case) was set based on the expected duration of vaccine immunity. In the case of low vaccine efficacy, it was assumed that the virus would spread in the part of the population with insufficient immunity, causing additional deaths and costs. However, due to the resulting immunity from vaccination and natural infection, future waves leading to a significant death toll and costs were not expected. While waning immunity from natural infections was discussed at the time of decision-making, reinfections causing a significant death toll and significant costs were generally not assumed [43]. Therefore, future waves were modelled only in the mitigation scenario. The transmission dynamics of SARS-CoV-2 were considered comparable to those of influenza [45], which typically causes epidemics in temperate climates every year during winter. In the absence of a vaccine, future COVID-19 pandemic waves were therefore assumed to peak in winter and return annually (yielding a total of five pandemic waves in the mitigation scenario).

#### Vaccine efficacy

Vaccine efficacy can be defined based on the attack rate (the proportion of individuals infected in a specific risk group over a nominated period) or the frequency of only severe cases [36]. The herd immunity threshold was calculated based on an inversely proportional relationship with vaccine efficacy measured in terms of attack rate [6]:

4 $$\varphi =\frac{1}{\epsilon}\left(1-\frac{1}{R_0}\right),$$

where *φ* refers to the herd immunity threshold, *ϵ* is vaccine efficacy, and *R*_0_ is the basic reproduction number of a disease.

#### Cost calculation

This study took a societal viewpoint, by including both direct medical costs and indirect/productivity costs. From the perspective of static efficiency the GDP drop associated with the lockdown/shutdown can be considered sunk at the time of decision-making. From the perspective of dynamic efficiency, which sets incentives for innovation (e.g., for vaccines in future pandemics), it is still relevant. As vaccine development and distribution in future pandemics is likely to occur only in conjunction with a shutdown strategy, considering the full shutdown cost avoids introducing excessive incentives for innovation. Therefore, a dynamic efficiency perspective was considered in the base case.

In the short term, a vaccination strategy must be regarded as an add-on to a mitigation strategy because vaccination of a large part of the population cannot be achieved immediately. However, mid- to long-term, vaccination avoids the costs of the mitigation strategy, which is the contribution of the lockdown/shutdown to the total economic burden of the COVID-19 pandemic. In addition, vaccination avoids deaths associated with mitigation strategy, which is not able to suppress the pandemic.

In terms of vaccination costs, the present study considered the costs of the vaccine itself, the clinical administration, subsidies, scientific research failures (in agreement with a dynamic efficiency perspective), vaccination centers, and transportation. In terms of the vaccine costs, this study considered prices that did not include a markup above the marginal costs. This is in line with the economic principle that drug prices need to be adjusted for producer surplus, as it presents a gain in societal welfare [22]. For the costs of scientific research failures, the probability of the success of clinical trials of vaccines was considered.

#### Willingness to pay

Willingness to pay (WTP) for an additional life-year was borrowed from new, innovative oncological drugs, as cancer is a condition with a similar perceived threat as COVID-19 [21]. In Germany, the prices of new, innovative oncological drugs are negotiated between the sickness funds and pharmaceutical companies. Even during these economically challenging times, past negotiation outcomes for pharmaceuticals are not revisited and hence are considered acceptable.

### Data

As mentioned in the introduction, the data used in the model and presented in the following are not the most recent. Nevertheless, they were relevant before the announcements of the COVID-19 vaccine results and hence were those that mattered for defining the EU vaccine procurement strategy. Table 1 presents the input values and distributions used in the base case and sensitivity analysis.

**Table 1:** Input values and distributions used in the base case and sensitivity analysis

Input	Mean (range)	Reference
*Epidemiological and clinical data*		
Population size by age	see reference	[14]
IFR in Germany	0.0075 (0.005 – 0.01)	[48]
CFR in Germany		[39–41]
Total population	0.042	
0-9 years	0.00009	
10-19 years	0.00005	
20-29 years	0.00022	;
30-39 years	0.00070	
40-49 years	0.0025	;
50-59 years	0.0095	
60-69 years	0.048	
70-79 years	0.16	
80-89 years	0.28	;
90+ years	0.33	
Probability of ICU indication	0.04 (0.04 – 0.08)	[39–41]
False-positive ICU admissions	0.1 (0.1 – 0.2)	[2]
CFR in the ICU	0.24 (0.23 – 0.25)	[39–41]
CFR one year post ICU discharge	0.59 (0.47 – 0.73)	[8]
Herd protection threshold	0.70 (0.60 – 0.70)	[28]
Vaccine efficacy	0.5 – 1.0	[17]
Immunity following one vaccination, years	5 (1 – 5)	[19, 30]
*Cost data*		
GDP reduction per pandemic wave, %	1.8	[12, 13]
GDP reduction without a second wave, %	5.0	[12, 13]
GDP drop attributable to shutdown versus voluntary restrictions, %	100 (10 – 100)	Estimate
German federal government subsidy for vaccine development	750,000,000	[16]
Contribution of a shutdown to GDP reduction, %	30 (10 – 30)	[38]
Vaccine costs per individual, €	8.47	[10]
Vaccination costs per individual, €	7.95	[29]

*CFR* case fatality rate, *ICU* intensive care unit, *IFR* infection fatality rate, *GDP* gross domestic product

#### Economic data

According to the European Economic Forecast by the European Commission in November 2020, Germany’s GDP was set to contract 5.5% in 2020. The second wave of infections was expected to dampen the rebound to 3.5% by 2021. Assuming that there was no permanent damage to productive capacity, Germany’s economy was projected to continue to grow above potential in 2022 at 2.5% and complete its recovery to the pre-crisis levels. As the 2021 GDP growth projection was revised to 3.5% from 5.3% in the forecast of July 2020, the impact of the second wave was calculated to be a 1.8% contraction of the GDP. This percentage was also applied to potential future waves. According to the European Economic Forecast, in Germany the total volume of the government measures “to fight the COVID-19 pandemic and stabilise the economy (…) amounts to 4.7% of GDP in 2020 and 2.1% in 2021”. GDP loss independent of the second wave was determined by subtracting the GDP contraction due to the second wave.

However, the European Economic Forecast was conducted assuming the absence of a pandemic in the counterfactual scenario, without considering the voluntary restrictions such as social distancing that may take place because of the rapid spread of the virus in the population [3]. In other words, individuals may take precautions even without lockdown orders. Accounting for the latter would decrease the incremental cost of the lockdown/shutdown over no pandemic. In the sensitivity analysis, the contribution of the lockdown/shutdown to the total loss of economic activities was assumed to be 10%, to account for the voluntary restrictions that may take place in the absence of a lockdown/shutdown. This estimate is in agreement with the one regarding the contribution of a shutdown to the loss of economic activities in Denmark, which was estimated to be 14% (=4%/29%) [42].

To determine the productivity gains resulting from a vaccination strategy compared with a mitigation strategy, I used the data sources reported in Table A1 of the Additional file 1.

The German federal government has funded three vaccine developers with a total of 750 million euros. BioNTech from Mainz received 375 million euros and CureVac from Tübingen received 230 million euros through a special vaccine development program [16]. This subsidy was included in the analysis and was related to one vaccinated individual.

Concerning the costs of the vaccine itself, an estimate of US$10 per person (converted to euros) was applied, which represents the costs of the Johnson & Johnson vaccine [10]. Johnson & Johnson declared that they did not to strive for profit [10]. The costs of failures were based on a failure rate of 70%, representing a weighted average of industry-sponsored and non-industry-sponsored vaccine development programs with end dates after January 1, 2000, and start dates before January 7, 2020 [47].

All costs are presented in euros (year 2020 values).

#### Clinical and epidemiological data

To calculate the per capita gain in life-years through mitigation, a COVID-19 infection fatality rate (IFR) of 0.75% [48], which was estimated in the summer of 2020, was applied to the previously developed model [21]. The IFR was adjusted upwards to account for the long-term mortality of ICU survivors. The per capita gain in life-years accounts for the percentage of the population that must be immune in order to reach the herd immunity threshold. Furthermore, given that the IFR is lower than the case fatality rate in Germany, the percentage of infected individuals admitted to the ICU was adjusted accordingly because a lower fatality rate also implies a lower percentage of cases admitted to the ICU [21].

According to the United States Food and Drug Administration (FDA) [17], the efficacy of the primary endpoint in a placebo-controlled efficacy trial should be at least 50% in order to classify a widely deployed COVID-19 vaccine as effective while ensuring safety. Hence, this estimate (50%) was taken as the lower limit of the vaccine efficacy. Because the FDA allows both SARS-CoV-2 infection and deaths associated with COVID-19 to be defined as primary endpoints, applying the 50% threshold to the life-years gained as a measure of vaccine efficacy is still valid.

If herd immunity is not reached due to low vaccine efficacy, local outbreaks may follow, necessitating local shutdowns/lockdowns. The economic costs of the latter were already accounted for by the economic projections in the absence of another pandemic wave, because the projections assumed continuous spreading of infections and only a “gradual lifting of containment measures” [12, 13].

In the base case, it was assumed that a vaccine campaign was able to overcome vaccine hesitancy by using strategies such as simple, easy-to-understand language [46]. Thus, the campaign was projected to achieve an uptake that is sufficient to yield herd immunity. Based on Eq. 4 and a herd immunity threshold of 70% for natural infection [28], the threshold is approximately 73% for a vaccine efficacy of 95%. For a vaccine efficacy of 50% the same uptake was assumed. In the sensitivity analysis, a vaccine uptake of 50% was considered based on a survey conducted in November 2020 in the German population [27].

Immunity was assumed to last between one [19] and 10 years. The latter estimate was based on the immunity status of survivors of severe acute respiratory syndrome, caused by another coronavirus, who still carry certain important immune cells 17 years after their recovery [30]. For comparison between vaccination and mitigation, the GDP drops associated with annual pandemic waves under mitigation were discounted at an annual rate of 3%, based on the social rate of time preference derived from the Ramsey equation [37]. For the health benefits of mitigation, a 2% discount rate was applied, reflecting a 1% expected growth rate of the consumption value of health in Germany [26].

#### Willingness to pay

The WTP for an additional life-year (€101,493 per life-year gained) was calculated by dividing the incremental costs of new, innovative cancer drugs (€39,751) by the incremental survival benefit (0.39 life-years) [20]. Because the WTP estimate does not account for life extension costs, the latter were not considered when calculating the cost-effectiveness of vaccination either (variable *b* in Eq. (2)).

## Results

A future lockdown policy avoids productivity losses due to symptomatic infections and quarantines of contact persons that are associated with an uncontrolled spread of the pandemic. Based on the results reported in Table A2 of the Additional file 1, the avoided productivity loss is predicted to be 0.9% of the GDP.

**Fig. 1:**
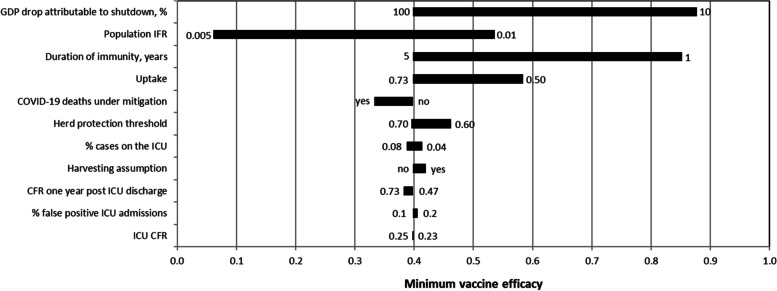
Tornado diagram demonstrating the results of the one-way sensitivity analysis. The variables are ordered by the impact on the minimum efficacy of a COVID-19 vaccine that makes vaccination cost-effective. The numbers indicate the upper and lower bounds

As stated below “Comparators”, the cost-effectiveness analysis compares a COVID-19 vaccination strategy with a mitigation strategy. The major driver of vaccination costs (€33, Table 2) is scientific research failures (€19). Vaccination with a vaccine with 50% efficacy followed by the lifting of mitigation measures is less effective than a long-term mitigation strategy. Nevertheless, it is still cost-effective because the savings are sufficiently large enough to pass the ICER threshold (Table 2). To determine the minimum vaccine efficacy (*e*_min_), Eq. 3 was applied. A vaccine must have an efficacy (*e*_min_) of at least 40% to be cost-effective in the base case. As shown in the sensitivity analysis (Fig. 1), the range of the minimum efficacy of a vaccine is between 6 and 88%. A small portion of the GDP loss attributable to the shutdown and a short duration of immunity have the largest impact on the minimum efficacy. Of note is the impact of lowering the herd immunity threshold, that is, when SARS-CoV-2 is less contagious. Although this improves the cost-effectiveness of lifting mitigation measures, vaccination becomes less valuable. This is because a lower herd immunity threshold translates ceteris paribus into a smaller contribution of vaccination to the total health benefits.

**Table 2:** Incremental costs, effects, and cost-effectiveness of a vaccine with efficacy 50% compared with a mitigation strategy. All costs are in Euro. Costs and life-years refer to one individual

Lockdown costs	Subsidy	Vaccination costs	Total costs	Life-years gained	ICER
-3159.89	9.02	33.32	-3117.55	- 0.02	128,353.85

*ICER* incremental cost-effectiveness ratio

## Discussion

This study re-analyzed the appropriateness of the EU’s COVID-19 vaccine procurement strategy in 2020 based on the cost-effectiveness criterion. The cost-effectiveness analysis compared vaccination followed the complete lifting of mitigation measures and a long-term mitigation strategy. The results reveal that the minimum COVID-19 vaccine efficacy against death that makes complete lifting cost-effective is 40% in the base case. The relatively high level of efficacy needed to demonstrate cost-effectiveness is supported by the sensitivity analysis, which shows considerable uncertainty around the minimum efficacy. Hence, even a vaccine efficacy level of 50% against death did not clearly justify the complete lifting of mitigation measures after vaccine rollout from a cost-effectiveness perspective. Similar concerns were raised from a public health perspective [33].

The minimum efficacy needs to be compared with the anticipated efficacy before the announcements of the phase III trial results. Many experts were expecting a vaccine efficacy of only 50 to 70% against symptomatic disease [50]. Therefore, given that the level of vaccine efficacy predicted by many experts did not clearly imply that lifting mitigation measures is cost-effective, the EU’s procurement strategy still appears to have been rational at the time of decision-making. This conclusion is supported by a UK field study conducted in the summer of 2020 that suggested an increase in vaccine hesitancy in view of lower efficacy [32]. A more aggressive order strategy seems to have been justifiable only for a rather optimistic decision maker, who, in a state of ambiguity, prioritizes upside potential over downside potential, thus deemphasizing potentially catastrophic events with low vaccine efficacy levels.

This decision-analytic study has several limitations. There are reasons why this study underestimates the health benefits and cost-effectiveness of a vaccine compared with a mitigation strategy and thus overestimates the minimum vaccine efficacy level. Some of these reasons have already been captured in the sensitivity analysis and include a low IFR. First, the study does not consider the deaths and loss of health-related quality of life associated with the shutdown and social distancing, for example, due to depressive or anxiety disorders, suicides, unemployment, domestic violence, and fewer emergency and regular visits to physicians due to unrelated medical conditions. Nevertheless, official data on excess mortality in Germany [15] showed that both excess mortality and COVID-19 mortality peaked in the first half of April 2020, indicating that excess mortality was driven by COVID-19 and not by other causes. Second, unaffected individuals may experience a loss of personal freedom [1] and autonomy under lockdown. Third, under the mitigation strategy elective procedures may need to be deferred if the ICU capacity is expected to be insufficient. Fourth, a vaccine may prevent COVID-19 infection with long-haul symptoms and reduces the direct (non-)medical and indirect costs associated with nonfatal COVID-19 cases.

Conversely, there are reasons to believe that the health benefits of mitigation and the minimum efficacy of a vaccine are underestimated. First, decreased economic activity can save lives, because it reduces air pollution, traffic accidents [7], and accidents at construction sites [9]. Second, social distancing may reduce the number of deaths caused by the flu. Third, despite criticism of underinvestment in vaccine procurement, the number of COVID-19 vaccines ordered by the EU has exceeded the required quantity, thus resulting in oversupply. Given the lack of information on the success rates of the various vaccines under development before the announcement of the first Phase III trial results, an oversupply could not have been predicted with certainty. Accounting for excess supply, however, would decrease the cost-effectiveness of a vaccine because of overspending and increase the minimum vaccine efficacy required to demonstrate cost-effectiveness. Some of the biases listed in this and the previous paragraph may cancel each other out.

Finally, the use of the number of life-years as an outcome measure may be criticized for lacking consideration of health-related quality of life. On the one hand, quality-adjusted life-years (QALYs) can capture additional health benefits resulting from the avoidance of non-fatal COVID-19 cases and the associated loss in quality of life. On the other hand, QALYs diminish the health benefits obtained from additional survival time by accounting for a quality-of-life decrement. As the QALY metric is thus biased against the elderly and the disabled, it has been considered ethically controversial [44]. For this and other reasons, QALYs have not yet been used in Germany for the purpose of reimbursing and pricing new, innovative medicines [25]. As another counterpoint, the public debate on COVID-19 in Germany before the trial announcements focused mainly on mortality as an endpoint and the number of life-years lost by the elderly who died from COVID-19. In sum, there is not a straightforward answer to the question of which outcome measure best reflects the value of a vaccine. Life-years gained may serve as a compromise between the use of unweighted lives saved and QALYs gained.

Although vaccines were ordered centrally by the EU, the cost-effectiveness of vaccination needs to be calculated from the viewpoint of each EU member state. The usual caveats apply in terms of the transferability and relevance of the analysis and conclusions from a German perspective to other countries. Specific reasons for caution include between-country differences in clinical and epidemiological data, costs, and willingness to pay for health benefits. Hence, low levels of vaccine efficacy may still be acceptable from the perspective of other EU member states.

To summarize, this study shows that at least part of the criticism of the EU’s COVID-19 vaccine procurement strategy does not appear to be justifiable in view of cost-effectiveness considerations and vaccine efficacy expectations before clinical trial announcements. The procurement strategy may not even be considered a failure from an ex post perspective. This is because mitigation measures have not been completely lifted since the summer of 2021 despite the wide availability of vaccines. The reason for this is new mutational variants. The EU procurement strategy can thus be seen as another example for the finding that “[t]he measures, recommendations and suggestions for managing the pandemic have not always followed a linear course, as they needed to be revised and updated as the scientific studies produced by the international scientific community provided increasing understanding and certainties about the virus” [31]. In retrospectively evaluating past policy decisions policymakers must be aware of hindsight bias, which may have played a role in the criticism of the EU’s COVID-19 vaccine procurement strategy after clinical trial announcements.

## Supplementary Information

**Additional file 1: Table A1.** Input data used for calculating the productivity loss due to an uncontrolled infection in the absence of a vaccine [4, 24, 34]. **Table A2.** Individual and population productivity loss due to an uncontrolled spread of the infection in the absence of a vaccine.

## Data Availability

All data are contained within the manuscript. The data sources are listed in Table 1 and are publicly available.
