# Supplementary material for: Was EU’s COVID-19 vaccine procurement strategy irrational? A re-analysis based on cost-effectiveness considerations
Source: BMC Health Serv Res. 2022 Nov 24;22:1410. doi: 10.1186/s12913-022-08726-4 (PMC9694594; doi:10.1186/s12913-022-08726-4)
Supplement: Supplementary file 1 — Additional file 1: Table A1. Input data used for calculating the productivity loss due to an uncontrolled infection in the absence of a vaccine [4, 24, 34]. Table A2. Individual and population productivity loss due to an uncontrolled spread of the infection in the absence of a vaccine. [file 12913_2022_8726_MOESM1_ESM.docx]

**Additional file 1**

Table A1. Input data used for calculating the productivity loss due to an uncontrolled infection in the absence of a vaccine.

| Input | Mean (range) | Reference |
| --- | --- | --- |
| *Epidemiological* | | |
| IFR in Germany | 0.0075 (0.005 – 0.01) | WHO 2020 |
| Percent of infections that are asymptomatic | 0.4 | Centers for Disease Control and Prevention 2020 |
| Percent of diagnosed infections that are asymptomatic | 0.38 | Robert Koch Institut 2020 |
| Percent of diagnosed infections that are hospitalized | 0.07 | Robert Koch Institut 2020 |
| Quarantined contact persons per diagnosed case | 5 | Grill 2020 |
| Number of newly diagnosed cases in August 2020 | 33,683 | Robert Koch Institut 2020 |
| *Cost data* | | |
| Hours worked per head and year in the population | 753.3 | OECD 2020 |
| Labor productivity per hour, € | 55.1 | OECD 2020 |

IFR = infection fatality rate

Table A2. Individual and population productivity loss due to an uncontrolled spread of the infection in the absence of a vaccine.

| Case description | Estimate | Per person productivity loss (€) | Population productivity loss (€) |
| --- | --- | --- | --- |
| Undiagnosed asymptomatic, % | 0.33 | 0 | 0 |
| Undiagnosed mild, % | 0.49 | 798 | 22,736,202,072 |
| Diagnosed asymptomatic, % | 0.07 | 798 | 3,154,064,309 |
| Diagnosed hospitalized, % | 0.01 | 1596 | 1,162,023,693 |
| Diagnosed mild, % | 0.10 | 798 | 4,565,093,078 |
| Quarantine of contacts, *n* | 1,178,905 | 798 | 941,011,720 |
| Total |  |  | 32,558,394,871 |
